# The scale-up cultivation of *Candida utilis* in waste potato juice water with glycerol affects biomass and *β*(1,3)/(1,6)-glucan characteristic and yield

**DOI:** 10.1007/s00253-018-9357-y

**Published:** 2018-09-13

**Authors:** Anna Bzducha-Wróbel, Katarzyna Pobiega, Stanisław Błażejak, Marek Kieliszek

**Affiliations:** Faculty of Food Science, Department of Biotechnolgy, Microbiology and Food Evaluation, Warsaw University of Life Sciences-SGGW, Nowoursynowska Str. 159c, 02-776 Warszawa, Poland

**Keywords:** *Candida utilis*, Cell wall, *β*-Glucan, Productivity, Agro-waste, Deproteinated potato juice water, Glycerol, Valorization, Osmotic stress

## Abstract

New ideas on production of yeast origin *β*-glucan preparations for industrial application are attracting interest considering market development of that high-value functional polysaccharide. Sellecting an efficient yeast producer and designing culture conditions are a prerequisite for obtaining high yield of *β*-glucan. The aim of this study was to describe at the first time the influence of the mode of cultivation (shake-flasks and batch fermentation) and time of culture on characteristic and yield of biomass and *β*(1,3)/(1,6)-glucan preparations of *Candida utilis* ATCC 9950 after cultivation in medium based on waste potato juice water supplemented with 10% of glycerol. After shake-flask culture, the biomass was characterized by higher protein content (app. 26.5%) compared to 19% after batch fermentation while the cultivation on a biofermentor scale promoted polysaccharides biosynthesis. The highest output of purified *β*(1,3)/(1,6*)*-glucan preparation (5.3 g_d.w._/L), containing app. 85% of that polysaccharide, was found after 48 h cultivation in biofermentor. Batch fermentation promoted biosynthesis of alkali-insoluble *β*(1,3)/(1,6)-glucan fraction, decreasing the content of *β*(1,6)-glucan. The yield of *β*(1,3)/(1,6*)*-glucan synthesis was 0.063 (g/g glycerol), while the productivity of that polysaccharide reached 0.094 (g/L/h). Longer batch fermentation (72 h) resulted in reduction of production efficiency of *β*-glucan preparation under studied conditions. The results of the study provide a new efficient biotechnological solution to produce high-value *β*-glucan preparations of *C. utilis* origin based on valorization of agro-waste potato juice water with glycerol.

## Introduction

There is an increasing biotechnological and industrial interest in the production of yeast origin *β*(1,3)/(1,6)-glucan for food, feed, pharmaceutical, cosmetics, and wastewater treatment applications (Zhu et al. 2016). That polysaccharide is used as a novel food component, natural immunomodulator, cholesterol-lowering substance, anti-cancer and anti-microbial agent, prebiotic, oral vaccine carrier, mycotoxin binder, wound healing substance, and ingredient of cosmetics (Auinger et al. 2013; Bacha et al. 2017; Baert et al. 2016; Borchani et al. 2016; Dalonso et al. 2015; Richter et al. 2015; Samuelsen et al. 2014; Shao et al. 2016; Stier et al. 2014; Zhu et al. 2016).

*β*(1,3)/(1,6)-Glucan is the main structural polymer of basket-like scaffold of yeast cell wall to which mannoprotein and chitin are cross-linked via *β*(1,6)-glucan chains (Gow et al. 2017; Klis et al. 2002; Lipke and Ovalle 1998). The chemical structure and concentration of that polysaccharide in yeast cell wall depend on genetic predisposition of the species and on environmental conditions of cells growth (Gow et al. 2017; Nguyen et al. 1998). Several cultivation factors, like the type and availability of carbon and nitrogen sources, cultivation temperature, pH, degree of aeration, osmotic pressure, the time of incubation and growth phase as well as mode of yeast propagation, influence the content and characteristic of structural polymers of yeast cell wall (Aguilar-Uscanga and François 2003; Bzducha-Wróbel et al. 2015; Jaehrig et al. 2008; Naruemon et al. 2013; Varelas et al. 2017). The influence of yeast *β*(1,3)/(1,6)-glucan chemical structure on biological activity and functional properties is still not precisely known (Batbayar et al. 2012; Wang et al. 2017).

Yeast cell wall integrity and strength are essential for cells survival under extreme environmental conditions (García et al. 2009; Gow et al. 2017). Stress factors that alter the cell wall architecture activate mechanisms of cellular response which adapt yeast cells so that they survive. The specific genes are expressed, mainly related to cell wall remodeling, metabolism, and signaling, leading to increase or decrease in *β*-glucan, chitin, or cell wall proteins contents (García et al. 2009; Gow et al. 2017; Varelas et al. 2017).

The influence of environmental factors on yeast cell wall composition, *β-*glucan content, and chemical characteristic of that polysaccharide is a present-day purpose of studies. There are only a few reports available explaining yeast cell wall remodeling under different growth conditions and even less consider the impact of mode and time of cultivation on the structure of the discussed organelle.

Industrially used yeast *β*-glucan preparations are currently isolated from the waste biomass of brewer’s yeast or from the biomass of baker’s yeast. The major contributors to the cost of biotechnological products are raw materials used for microbial cultivation (Ferrari et al. 2001). Yeast can be easily cultivated in diverse types of growth media. The biomass of food grade yeasts is mainly produced utilizing conventional substrates like molasses, a by-product from sugar industry, but nowadays it becomes a scarce resource. Starch, distiller’s wash, whey, fruit and vegetable wastes, and unconventional materials, like petroleum by-products, are also applied (Bekatorou et al. 2006). The industrial competitiveness in yeast *β*-glucan production could be improved by application of new, efficient yeast species as a biosynthesis system of that polysaccharide but also by replacement of molasses with cheap and locally available sources of nutrients for yeast cultivation. Deproteinated potato juice water (DPJW) is a by-product in the production of potato starch (Dzwonkowski 2012). The waste is not valorized nowadays while its utilization in the production of functional microbial metabolites as culture medium is possible (Bzducha-Wróbel et al. 2015). The chemical composition of DPJW confirms its usefulness as a source of nitrogen, mineral compounds (potassium, sulfur, phosphorus, sodium, calcium, magnesium, and manganese), as well as vitamins (C, B_1_, B_2_, B_6_, PP, and E), all essential for yeast growth (Kowalczewski et al. 2012; Kurcz et al. 2018). The increase in the content of organic carbon source in DPJW is required for efficient productivity of cellular biomass. For this purpose, it is possible to use glycerol (Bzducha-Wróbel et al. 2015). Many yeast species are able to utilize glycerol as a carbon and energy source, including crude glycerine fraction from biodiesel production (Chiruvolu et al. 1999; Kurcz et al. 2018). Glycerol is a precursor for important cellular components and compatible solute allowing cells to respond quicly to changes in osmolarity (Rivaldi et al. 2008). However, there are still gaps in our knowledge of glycerol metabolism and transport in yeast (Klein et al. 2017).

The results of our previous studies (Bzducha-Wróbel et al. 2015) showed that cultivation of *Candida utilis* ATCC 9950 yeast strain in media with DPJW supplemented with glycerol contributes to important intensification (app. 45%) of *β*(1,3)/(1,6)-glucan synthesis in cell walls of that strain. This was the basis for development of a culture aimed at *C. utilis* biomass production with an increased content of *β*-glucan with simultaneous utilization of agro-food industry waste*.* The *C. utilis* species is recognized as safe, being commercially used in the production of food additives and nutritional feed supplements for more than 70 years (Bekatorou et al. 2006; Rosma and Ooi 2006).

The current study aimed to describe the effect of cultivation mode (shake-flasks and batch fermentation) and time of *C. utilis* ATCC 9950 propagation in waste potato juice water with 10% of glycerol on characteristic and productivity of biomass and *β-*glucan preparations. For this purpose, yeasts were grown in shake-flasks (72-h culture) and at the scale of laboratory 5 L-biofermentor (48- and 72-h cultures).

Presented results are useful for industrial production of yeast origin *β*-glucan preparations but also deepen the knowledge about biosynthesis of cell wall polysaccharides and glycerol metabolism in yeast.

## Materials and methods

### Yeast strain

The yeast strain of *C. utilis* ATCC 9950, collected in the Museum of Pure Cultures at Division of Food Biotechnology and Microbiology, Faculty of Food Science, Warsaw University of Life Sciences-SGGW, was studied as a *β*-glucan source. Yeast culture was stored at 4 °C on agar slants of YPD medium (BTL, Poland).

### Cultivation medium

The cultivation medium was composed of DPJW with 10% (*w*/*v*) of glycerol as a carbon source and pH 5.0 ± 0.2. Waste DPJW was obtained from the processing line of company producing potato starch (Mazovia region, Poland). The medium was sterilized at 121 °C/0.1 MPa/20 min (HICLAVE HG80 autoclave, Hirayama, Japan) and characterized according to Bzducha-Wróbel et al. (2015) considering the content of dry substance (drying-weighing method at 105 °C/24 h), total organic carbon (TOC; high-temperature oxidizing method using IL 550 TOC-TN analyzer), total nitrogen content (Kjeldahl method, BÜCHI mineralization and distillation units), and directly reducing sugars (colorimetric method with 3.5-dinitrosalicylic acid). Furthermore, contents of selected elements in DPJW were determined (K, S, P, Na, Ca, Mg, Mn) by ICP technique in atomic emission spectrometer (ICP-AES Thermo iCAP 6500 DUO). Obtained results were stated in grams per 1 L of the medium and presented in Table 1.

**Table 1:** Chemical composition of deproteinated potato juice water

Component	Unit	Value
Dry matter	(g_d.w./_L)	35.1 ± 0.3
Total organic carbon	(g/L)	15.6 ± 0.0
Sugars		7.8 ± 0.2
Nitrogen		2.2 ± 0.2
Protein		13.9 ± 0.1
Potassium		7.4 ± 0.058
Sulfur		5.9 ± 0.96
Phosphorus		0.691 ± 0.06
Sodium		0.258 ± 0.09
Calcium		0.418 ± 0.06
Magnesium		0.582 ± 0.04
Manganese		0.003 ± 0.00

### Inoculum

Liquid YPD medium (BTL, Poland) was used for cultivation of yeast *inoculum* (four flasks with 80 cm^3^ of medium, 500 cm^3^ flask). The medium was inoculated with yeast cells from the slant cultures. The cultures were grown at 28 °C for 24 h with shaking (200 cycles/min, SM-30 Control Buechler, Germany). After incubation, yeast biomass was separated from the culture medium by centrifugation (3900×*g*/10 min., Eppendorf, 5804R, Germany), rinsed with sterile water, centrifuged, and resuspended in 80 cm^3^ of DPJW with 10% of glycerol (Avantor Performance Materials, Poland). Obtained material constituted *inoculum* for proper culture experiments in flasks and biofermentor scale.

### Mode of cultivation and conditions

#### Shake-flask culture

Shake-flask cultivations of studied *C. utilis* yeast were conducted in 500 cm^3^ flasks containing 90 cm^3^ of sterile cultivation medium. The cultures were inoculated using 10% (*v*/*v*) of *inoculum.* Yeast were cultivated during 72 h at 28 °C with the rate of shaking 200 cycles/min (SM-30 Control, Buechler, Germany). Three parallel cultures were carried out in the flasks. Two cultures were run in parallel with 72 h cultures in the biofermentor. One further culture was carried out using an inoculum that was prepared to initiate 48 h cultivation in the biofermentor. One breeding means multiplication in three parallel flasks. Biomass from parallel flasks was averaged, which allowed to obtain enough material to carry out the scope of the research. The same batch of the waste potato juice water was used to carry out all the cultivations.

#### Biofermentor scale culture

Cultivation at scale-up mode was performed in batch 5-L fermentor (BIOFLO 3000, New Brunswick, USA) with the working volume of 3 L. The growth medium (2.7 L) was inoculated using 10% (*v*/*v*) of *inoculum*. The impeller rotation speed was 300 rev/min, temperature was kept at 28 °C, and airflow at 2.5 L/min. Foam was controlled using Acepol 7287 antifoam (Dakis-Biotimex, Poland). The cultivation times were 48 and 72 h. Two independent cultivations were performed for each growing time. Each cultivation was carried out using another inoculum but the same batch of waste potato juice water.

### Cell mass production

Cell mass production after yeast cultivation at experimental conditions was determined by drying-weighting method according to Bzducha-Wróbel et al. (2015). The results of cell mass production were given in grams of dry weight per liter of culture medium (g_d.w._/L). After yeast cultivation at experimental conditions, the biomass was collected by centrifugation (3900×*g*/4 °C/10 min, Eppendorf 5804R, Germany). Biomass specimens were rinsed three times with water and centrifuged each time. Two parts of biomass from each culture conditions were used for yeast cell wall preparation. One part of biomass was lyophilized to obtain freeze-dried preparation for further chemical characterization.

### Cell wall preparations

Cell wall preparations were produced by mechanical disintegration of yeast biomass in Bead-Beater GB26 (Biospec Products Inc., USA) bead mill according to Bzducha-Wróbel et al. (2015). The preparations were lyophilized and milled following the methodology described for freeze-drying. The material obtained in that way constituted impurified cell walls that were used for *β*(1,3)(1,6)-glucan isolation and further analysis.

### Isolation of *β*(1,3)(1,6)-glucan preparations

The procedure of *β*(1,3)/(1,6)-glucan isolation and purification was performed on the basis of methods recommended by Freimund et al. (2003) and Magnani et al. (2009) with modifications. Approximately 2800 mg of cell wall preparations was weighted and suspended in 0.02 M sodium potassium buffer, pH 7.5 (Avantor Performance Materials, Poland, Gliwice). Zirconium-glass beads of 1 mm in diameter (Biospec Products Inc., Bartlesville, OK, USA) were added and samples were autoclaved (121 °C/0.1 MPa/30 min). Next, all samples were centrifuged (4600×*g*/4 °C/10 min) and obtained specimens were rinsed with water three times and centrifuged each time. Then, 11.2 cm^3^ of isopropyl alcohol was added to specimens (Avantor Performance Materials, Poland) and samples were incubated in water bath with shaking (60 °C/2 h, Memmert WNB14, Germany). Supernatants were poured out while specimens were three times rinsed in deionized water. The enzymatic digestion of proteins present in specimens of cell walls preparations was aided by pronase E enzyme (Sigma-Aldrich, USA) using 200 ng/cm^3^ of enzyme solution prepared in 0.01 M potassium phosphate buffer (pH 7.0) with the addition of 5 mg/cm^3^ of sodium lauryl sulfate (Avantor Performance Materials, Poland). The sludges of purified preparations were suspended in 35 cm^3^ of enzyme solution. Samples were incubated at 37 °C for 24 h with shaking (Memmert WNB14, Germany). After enzymatic hydrolysis, specimens were rinsed in water three times, centrifuged, lyophilized, and milled. Obtained purified *β*-glucan preparation was characterized in compliance with points regarding chemical composition of produced preparations.

### Lyophilization

The process of lyophilization of *C. utilis* yeast biomass, yeast cell wall preparation, and purified *β*-glucan preparations was carried at Christ Freeze Dryer Alpha 1-4 LSC plus apparatus (Germany). The drying program was selected experimentally. The pressure was set at 0.5 mPa, and the initial temperature of the main drying was set at − 25 °C, while the final temperature was 5 °C. At the beginning of the process, the temperature was raised by 5 °C every 5 h, and then every 2 h. The process lasted 18 h. After this time, additional drying process was carried out at 10 °C during app. 2 h. After lyophilization, the obtained preparations were grinded and stored for further determinations.

### Post-culture medium characterization

#### Potentiometric acidity

The pH values of culture mediums before and after yeast cultivation were determined using CP-505 ELMETRON pH-meter (Poland).

#### Total sugar content

The content of total sugar in culture mediums (before and after yeast cultivation) was determined as glucose-equivalent reducing sugars after acid hydrolysis of the medium samples (1 cm^3^, in triplicate for each cultivation type). Acid hydrolysis was performed using 13.5 M H_2_SO_4_ (Avantor Performance Materials, Poland) at 95 °C for 4 h. The content of saccharides was calculated using a standard curve prepared for glucose (y = 2.3279x − 0.0755 (mg/ cm3), R^2^ = 0.9989) obtained by the colorimetric method with 3,5-dinitrosalicylic acid (Sigma-Aldrich, USA) at λ = 540 nm (SmartSpec 3000 Bio-Rad Laboratories Inc., USA).

#### Glycerol content

Glycerol content in mediums before and after cultivation was determined using method proposed by Milchert et al. (1995) involving oxidizing activity of meta-periodic acid (Chempur, Poland) to hydroxyl groups of glycerol.

#### Nitrogen content

Total nitrogen content in analyzed media (before and after cultivation) was determined with the Kjeldahl method (Büchi mineralization and distillation units, Büchi Labourtechnik, Switzerland) after mineralization of 5 cm^3^ of each sample (in triplicate for all cultivation conditions—72 h of cultivation in flasks, 48 and 72 h of cultivation in biofermentor).

### Calculation of production parameters of biomass, cell wall, *β*-glucan preparations, and *β*(1,3)/(1,6)-glucan

Production parameters of *C. utilis* ATCC 9950 cell biomass, cell wall, *β*-glucan preparations, and *β*(1,3)/(1,6)-glucan polymer in relation to mode and time of cultivation were calculated according to definitions presented by Sitepu et al. (2014). Cell mass yield was expressed as grams of dry weight of yeast biomass per grams of glycerol consumer (g_d.w._/g glycerol) after proper cultivation time at flask and biofermentor scale.

Parameters of cell wall and *β*-glucan preparations outputs (volumetric productivity) were expressed in grams per liter of culture medium based on the mass of obtained preparations. The output of *β*(1,3)/(1,6)-glucan (based on the *β*(1,3)/(1,6)-glucan concentration in purified *β*-glucan preparations) was calculated as grams of glucan per liter of culture medium. Yield of *β*(1,3)/(1,6)-glucan was calculated as grams of glucan per grams of glycerol consumed during cultivation. The productivity parameter of *β*(1,3)/(1,6)-glucan was expressed as grams of mentioned polysaccharide per liter of culture per hour of cultivation. The specific rate of *β*(1,3)/(1,6)-glucan formation was estimated as grams of mentioned polysaccharide per gram of yeast biomass dry weight per hour of cultivation.

### Chemical characterization of yeast biomass, cell walls, and *β*-glucan preparations

#### Protein content

Total nitrogen content in the analyzed samples of yeast biomass, cell walls, and *β*-glucan preparations was determined with the Kjeldahl method (Büchi mineralization and distillation units, Büchi Labourtechnik, Flawil, Switzerland) after mineralization of 200 mg of each sample of preparations from experimental condition. Nitrogen content was expressed per crude proteins using a conversion factor of 6.25.

#### Total saccharides and *β*(1,3)(1,6)-glucan content

The content of total saccharides as reducing sugars (expressed per glucose) was determined with the colorimetric method using DNS (point 2.9.2). Before determination, cell wall polymers (app. 20 mg) were subjected to acidic hydrolysis (13.5 M H_2_SO_4_, 95 °C/4 h) in water bath (Memmert WNB14, Germany).

The total content of *β*(1,3)/(1,6)-glucan was determined using an Enzymatic Yeast Beta-Glucan Kit (K-EBHLG, Megazyme, Ireland) following procedure recommended by the producer. The UV-1800 UV/VIS, Rayleigh (China) spectrophotometer was used.

#### The content of alkali-soluble and alkali-insoluble polysaccharide fractions in purified *β*-glucan preparations

Obtained preparations, rich in *β*(1,3)/(1,6)-glucan, were characterized in terms of the content of alkali-soluble and alkali-insoluble polysaccharides fractions (Bzducha-Wróbel et al. 2013). Approximately 20 mg of purified *β*-glucan preparations was taken. Next 1.5 cm^3^ of 3% NaOH (Avantor Performance Materials, Poland) was added in order to run the first cycle of alkaline extraction in the water bath (75 °C/1 h). Subsequent samples were centrifuged (3900×*g*/10 min) and supernatants were collected. Additional 1.5 cm^3^ of NaOH portion was added to each specimen obtained after first extraction step and samples were incubated under conditions as described above. After incubation samples were centrifuged and the obtained supernatants were mixed with the alkali-soluble fraction collected after the first step of extraction. The last cycle of extraction was performed using 2.0 cm^3^ of NaOH added to each specimen. Finally, supernatants derived from three subsequent stages of alkaline extraction of a given sample were combined and analyzed in terms of the total content of polysaccharides soluble in alkali (soluble *β*(1,3)/(1,6)-glucan fractions, mannoproteins, α-glucan). For that purpose, all samples were subjected to acid hydrolysis and total sugars were measured by colorimetric method with DNS as described for total sugar analysis.

The specimens derived by alkaline extraction contain yeast *β*(1,3)- and *β*(1,6)-glucan fractions insoluble in alkali—the main *β*-glucans type present in yeast cell wall. The total content of alkali-insoluble *β*-glucans was determined by means of total sugars analysis by colorimetric method with DNS after acid hydrolysis of specimens. At the same time specimens of polysaccharides insoluble in alkali (parallel extractions) were subjected to enzymatic hydrolysis using Zymolyase 20T preparation (MP Biomedicals LLC, USA) according to the point of the methodology described below.

#### The content of *β*(1,3)- and *β*(1,6)-glucan insoluble in alkali

Specimens of *β*-glucan fractions insoluble in alkali were subjected to enzymatic digestion using Zymolyase 20T preparation (MP Biomedicals LLC, USA) (Bzducha-Wróbel et al. 2013). The preparation consists in *β*(1,3)-glucan laminaripentaohydrolase, *β*(1,3)-glucanase, protease, mannanase, amylase, xylanase, and phosphatase. The glucose polymers are hydrolyzed at the *β*(1,3)-glucan linkages with laminaripentaose as the principal product while *β*(1,6)-glucan is not subjected to digestion. The purpose of this stage was to define the content of alkali-insoluble *β*(1,3)- and *β*(1,6)-glucan individually. The content of *β*(1,3)-glucan was calculated as difference between the total content of sugars present in the alkali-insoluble specimen derived after alkali extraction and total content of *β*(1,6)-glucan (sum of sugars analyzed in specimen derived by zymolyase hydrolysis of alkali-insoluble fraction plus sugars analyzed in samples dialysates of after enzyme digestion). It was assumed that alkali-insoluble specimens do not contain chitin.

Alkali-insoluble specimens were rinsed with 0.1 M Tris-HCl (Sigma-Aldrich, USA) buffer (pH 7.4) twice and then centrifuged (3214×*g*/15 min). Next 1.5 cm^3^ of zymolyase preparation (5 mg/cm^3^) dissolved in 0.01 M Tris-HCl buffer (pH 8.0) was added to each sample. Samples were incubated in water bath (20 h) with shaking and at the temperature of 37 °C. After hydrolysis, samples were centrifuged (3214×*g*/15 min). Obtained specimens contained *β*(1,6)-glucan fractions not digested by the enzyme. The content of *β*(1,6)-glucan was estimated as total sugars by colorimetric method with DNS after acid hydrolysis of specimens.

The content of *β*(1,6)-glucan was also defined in dialysates of supernatants derived after zymolyase digestion of specimens insoluble in alkali. Dialysis was conducted in order to separate fractions of hydrolyzed *β*(1,3)-glucan from non-hydrolyzed *β*(1,6)-glucan remained in dialysates. Dialysis was carried out using high retention cellulose tubing bags (Sigma-Aldrich, USA) submerged in deionized water for 24 h. Samples were placed on the magnetic stirrer (ES 24, WIGO, Poland). Next, dialysates were subjected to acid hydrolysis and total sugars were determined by colorimetric method with DNS.

### Analysis of yeast cell wall using transmission electron microscopy

The analysis of cell wall structure after studied yeast cultivation was performed using transmission electron microscopy (TEM). Samples were prepared according to procedure described by Bzducha-Wróbel et al. (2015).

### Statistical analysis

Obtained results were subjected to a statistical analysis using the STATISTICA V.13.1 program. An analysis with the ANOVA method (Tukey’s test) was carried out at the *α* = 0.05 level of significance.

## Results

### Cell mass production, biomass chemical characteristic, and nutrients utilization in relation to the mode of cultivation

Production parameters of *C. utilis* ATCC 9950 cell biomass were presented in Table 2. After 72 h of cultivation in shake-flasks, about 25 g of dry weight (dry cell matter) per liter of medium was obtained. Batch fermentation contributed to the increase in the biomass production to app. 31–32 g_d.w._/L (48 and 72 h, respectively).

**Table 2:** Production parameters of *C. utilis* ATCC 9950 cell biomass, cell wall, *β*-glucan preparations, and *β*(1,3)/(1,6)-glucan depending on scale and time of cultivation

	Output (volumetric productivity)				Yield		*β*(1,3)/(1,6)-Glucan productivity**	Specific rate of *β*(1,3)/(1,6)-glucan formation**
Type of culture	Cell mass production	Cell wall preparation	*β*-Glucan preparation	*β*(1,3)/(1,6)-Glucan **	Cell mass yield	*β*(1,3)/(1,6)-Glucan yield**		
		(g_d.w./_L)			(g_d.w._/g glycerol consumed)		(g/L/h)	(g *β*-glucan/g_d.w._biomass/h)
*Flask/72h	25.6 ± 0.8^a^	6.4 ± 0.6^a^	3.5 ± 0.9^a^	2.8	0.383	0.042	0.039	0.0015
Biof/48h	31.3 ± 0.5^b^	8.1 ± 0.5^b^	5.3 ± 0.8^b^	4.5	0.440	0.063	0.094	0.0030
Biof/72h	32.2 ± 1.9^b^	8.4 ± 0.9^b^	5.1 ± 0.7^b^	4.4	0.423	0.058	0.061	0.0019

*Flasks/72h—cultivation in flask during 72 h; Biof/48h and Biof/72h—cultivations in biofermentor adequately 48 and 72 h; ** based on the *β(1,3)/(1,6)*-glucan concentration in purified *β*-glucan preparations; a, b, c…—mean values marked with the same letters do not differ significantly, Tukey’s test, *α* = 0.05. Each value represents the mean and standard deviation from three independent experiments in flask and two independent cultures in biofermentor for each of studied time of cultivation. Replicates were done on different days with different inocula stocks, using the same batch of deproteinated potato juice water as a medium

**Fig. 1:**
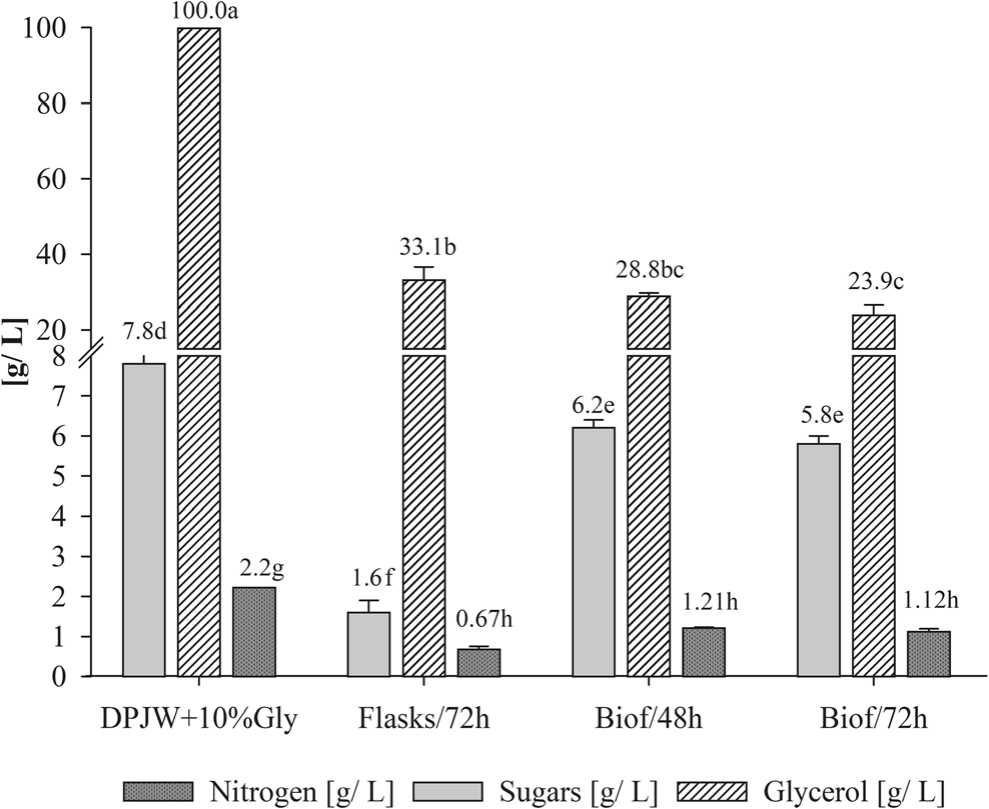
Changes in glycerol, sugars, and nitrogen contents in cultivation mediums depending on the mode and time of cultivation (a, b, c…—mean values marked with the same letters do not differ significantly, Tukey’s test, *α* = 0.05; Flasks/72h—72 h culture in shake-flasks, Biof/48h and Biof/72h—batch fermentation cultures after 48 and 72 h; DPJW+10%—composition of culture medium at time “0”). Each value represents the mean and standard deviation from three independent experiments in flask and two independent cultures in biofermentor for each of studied time of cultivation. Replicates were done on different days with different inocula stocks, using the same batch of deproteinated potato juice water as a medium

The results of biomass production correlated with glycerol utilization under studied conditions. After 72 h of batch fermentation, glycerol consumption reached about 76% while in shake-flasks it was app. 67% (Fig. 1, Table 3). Cell mass yields of the studied *C. utilis* yeast strain amounted to 0.423–0.440 g_d.w._ biomass per 1 g of utilized glycerol after 72 and 48 h of culture in biofermentor (respectively). In flasks it was 0.383 g_d.w._/g of glycerol (Table 2).

**Table 3:** Utilization of glycerol, sugars, and nitrogen during cultivation of *C. utilis* ATCC 9950 on potato waste water with glycerol and pH changes depending on the scale and time of culture (%)

Type of culture	Glycerol	Sugars	Nitrogen	pH**
	(%)			
*Flask/72h	66.9^a^	79.5^c^	69.7^c^	6.9
Biof/48h	71.2^b^	20.5^a^	45.0^a^	7.1
Biof/72h	76.1^c^	25.6^b^	49.1^b^	7.4

*Flasks/72h—cultivation in flask during 72 h; Biof/48h and Biof/72h—cultivations in biofermentor adequately 48 and 72 h; ** initial pH of culture medium was 5.0 for each culture; a, b, c…—mean values marked with the same letters do not differ significantly, Tukey’s test, *α* = 0.05. Each value represents the mean and standard deviation from three independent experiments in flask and two independent cultures in biofermentor for each of studied time of cultivation. Replicates were done on different days with different inocula stocks, using the same batch of deproteinated potato juice water as a medium

In tested media consisting of DPJW and glycerol, the glucose-equivalent reducing sugars were also available, however at low concentration (app. 7.8 g/L). Sugars were also utilized by examined yeast cells as carbon and energy source (Fig. 1). Significant differences between the type of culture and the degree of sugars uptake were noticed. The highest use of that nutrients (about 79.5%) was found after 72 h of *C. utilis* cultivation in the flasks, while a definitely lower level of sugar utilization was observed during batch fermentation (20–26%, after 48 and 72 h, respectively) where glycerol assimilation was preferential at the same time (Table 3).

The availability of nitrogen source in the culture medium is a limiting factor for microbial cells growth and synthesis of cellular proteins. Initial nitrogen content in DPJW was approx. 2.2 g/L (Fig. 1). Depending on the scale of *C. utilis* propagation, different nitrogen utilization rates in DPJW were observed. *C. utilis* cultivation on biofermentor scale for 48 h resulted in 45% consumption of initially available nitrogen while additional 24 h of incubation caused 49% utilization (Table 3). Nearly 70% of initially available nitrogen was used by yeasts grown in flasks.

**Fig. 2:**
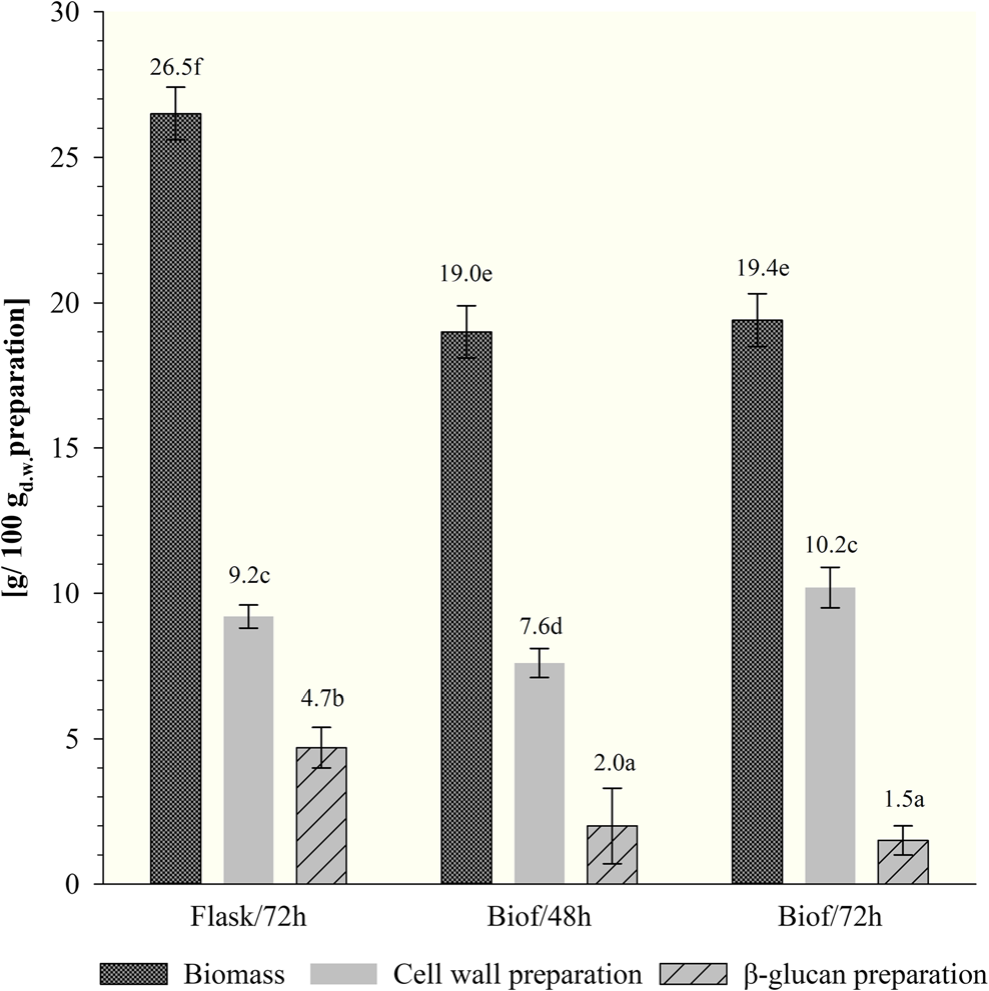
The content of proteins in biomass, cell wall, and purified *β*(1,3)/(1,6)-glucan preparations of *C. utilis* ATTC 9950 depending on the mode and time of cultivation in deproteinated potato juice water with 10% of glycerol (a, b, c…—mean values marked with the same letters do not differ significantly, Tukey’s test, *α* = 0.05; Flasks/72h—72 h culture in shake-flasks; Biof/48h and Biof/72h—batch fermentation cultures after 48 and 72 h). Each value represents the mean and standard deviation from three independent experiments in flask and two independent cultures in biofermentor for each of studied time of cultivation. Replicates were done on different days with different inocula stocks, using the same batch of deproteinated potato juice water as a medium

The biomass propagated in biofermentor was characterized with significantly lower protein content (19%, regardless of the time of multiplication) comparing with cells from shake-flask system (26.5%) (Fig. 2). The results indicate the flasks mode of cultivation favored intracellular protein synthesis, what explain the higher nitrogen utilization in that variant of yeast cultivation. Considering production process of *β*-glucan preparations isolated from yeast biomass, high intracellular protein content is not desirable due to the requisite purification processes.

**Fig. 3:**
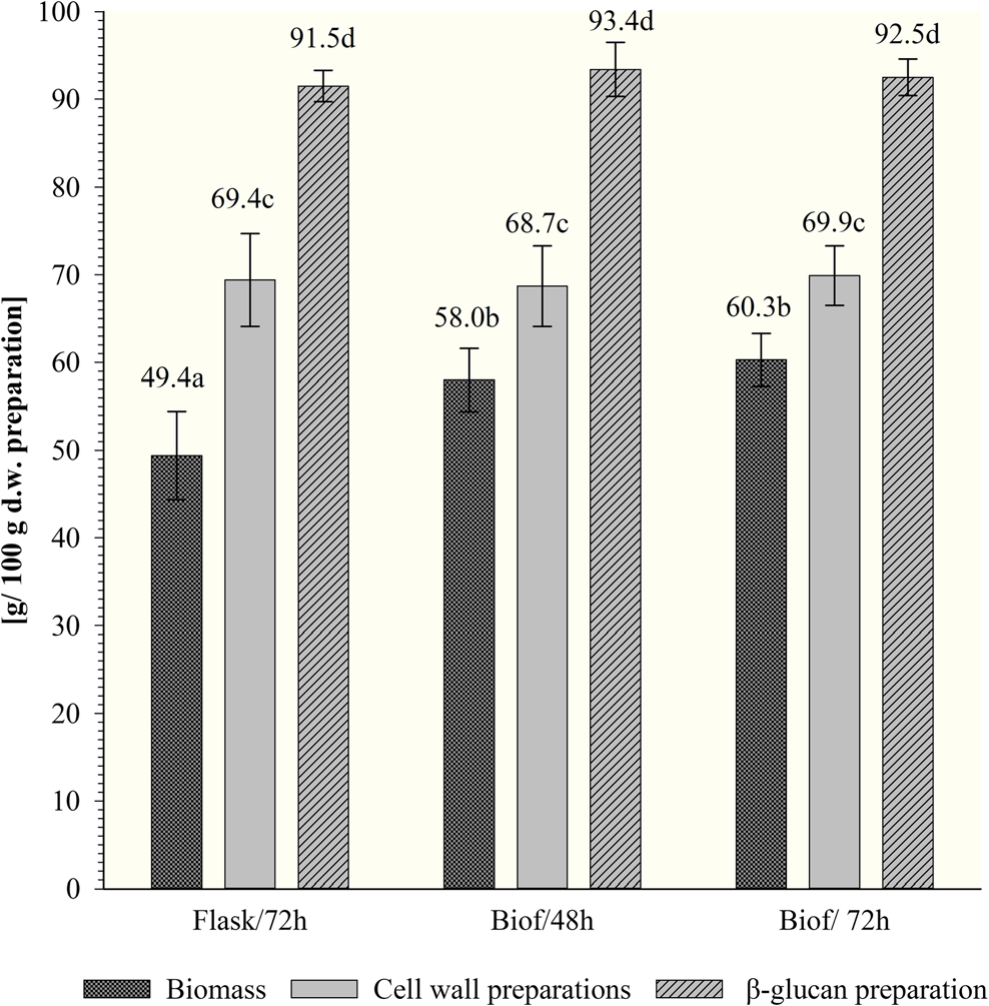
The content of total sugars in biomass, cell wall, and purified *β*(1,3)/(1,6)-glucan preparations of *C. utilis* ATTC 9950 depending on the mode and time of cultivation in deproteinated potato juice water with 10% of glycerol (a, b, c…—mean values marked with the same letters do not differ significantly, Tukey’s test, *α* = 0.05; Flasks/72h—72 h culture in shake-flasks; Biof/48h and Biof/72h—batch fermentation cultures after 48 and 72 h). Each value represents the mean and standard deviation from three independent experiments in flask and two independent cultures in biofermentor for each of studied time of cultivation. Replicates were done on different days with different inocula stocks, using the same batch of deproteinated potato juice water as a medium

The preparations of biomass of tested *C. utilis* yeast were characterized in terms of total sugars concentration (Fig. 3). Depending on the mode of cultivation, significant differences in the content of total sugars in biomass of examined yeast were found. The lowest sugars concentration was determined in cells propagated in shake-flasks (about 49%), while definitely higher (about 60%) was observed in biomass propagated in biofermentor.

Changes in pH of culture mediums during yeast growth indicated alkalization (Table 3), occurring to a greater extent on the biofermentor scale. The potential acidity was intentially not stabilized during batch fermentation to compare with flask-shake cultures.

### Production parameters of cell wall and *β*(1,3)/(1,6)-glucan preparations

The parameters related to the productivity of cell wall preparations and *β*(1,3)/(1,6)-glucan of *C. utilis* ATCC 9950 yeast were shown in Table 2. The output of tested preparations was expressed in grams of dry weight of cell walls or *β-*glucan achieved from 1 L of culture (volumetric productivity). The highest outputs of cell wall preparations (app. 8.1 g_d.w._/L) and purified *β*-glucan preparations (5.3 g_d.w._/L) were noted after 48 h of batch fermentation. The yield of *β*(1,3)/(1,6)-glucan biosynthesis, determined on the basis of this polysaccharide content in purified preparations and expressed in grams per grams of glycerol utilized, amounted to 0.063 g/g of glycerol. The productivity of *β*(1,3)/(1,6)-glucan was 0.094 (g/L/h) while the specific rate of *β*(1,3)/(1,6)-glucan biosynthesis amounted to 0.0030 (g/g_d.w._ biomass/h). Discussed mode and time of *C. utilis* ATCC 9950 cultivation was found to be the most beneficial for conducting the biosynthesis process of *β*(1,3)/(1,6)-glucan under given culture conditions. After longer incubation time on biofermentor scale (72 h), all production parameters related to *β*(1,3)/(1,6)-glucan were lower comparing with 48 h culture. The lowest production parameters of cell wall and *β-*glucan preparations were observed in shake-flasks culture.

### Chemical characteristic of cell walls and purified *β-*glucan preparations in relation to the mode and time of yeast cultivation

#### Protein content

Figure 3 presents the results of protein content in cell wall preparations and *β*-glucan preparations of tested *C. utilis* yeast, depending on the culture variant. Isolation of cell wall preparations from the biomass cultivated in shake-flask resulted in reduction of proteins content from 26.5% in biomass to about 9.2%. Cell wall preparations obtained from yeast propagated in batch fermentation consist with 7.6 and 10.2% of protein, after 48 and 72 h, respectively. The protein content in purified *β*-glucan preparations was reduced to about 5%, when they were isolated from cells cultivated in shake-flasks. About 1.5–2% of protein was stated in *β*-glucan preparations isolated from biomass obtained after batch fermentation.

#### Total sugars and *β*(1,3)/(1,6)-glucan content

**Fig. 4:**
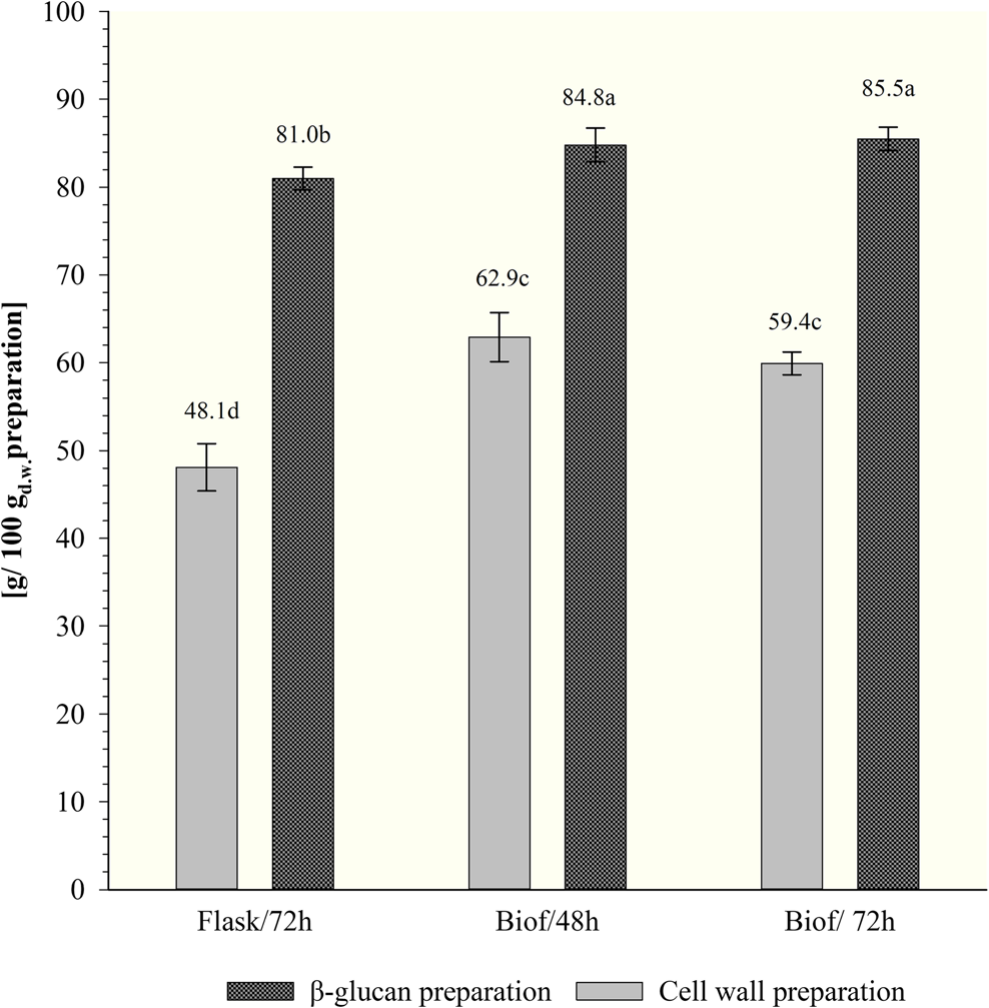
The content of *β*(1,3)/(1,6)-glucan in cell wall and purified *β*(1,3)/(1,6)-glucan preparations of *C. utilis* ATTC 9950 depending on the mode and time of cultivation in deproteinated potato juice water with 10% of glycerol (a, b, c…—mean values marked with the same letters do not differ significantly, Tukey’s test, *α* = 0.05; Flasks/72h—72 h culture in shake-flasks, Biof/48h and Biof/72h—batch fermentation cultures after 48 and 72 h). Each value represents the mean and standard deviation from three independent experiments in flask and two independent cultures in biofermentor for each of studied time of cultivation. Replicates were done on different days with different inocula stocks, using the same batch of deproteinated potato juice water as a medium

The results of total sugar content in cell walls and *β*-glucan preparations were presented on Figs. 3 and 4. The content of sugars in the wall preparations was estimated at about 69%, regardless of the initial intracellular sugar (Fig. 3) and protein contents in the biomass studied. It confirms efficient releasing of intracellular components during cell wall preparation. In purified *β*-glucan samples, sugars amounted to approx. 91.5–93.4% of dry matter, after 72 h of culture in flasks and 48 h incubation in biofermentor, respectively.

The content of *β*(1,3)/(1,6)-glucan was determined only in cell walls and purified *β*-glucan preparations (Fig. 4). The use of an enzymatic assay based on hydrolytic activity of *exo*- and *endo*-*β*(1,3)-glucanase to determine the content of this polysaccharide in yeast biomass directly is difficult and burdened with error. Structural *β*-glucans of yeast cell wall are cross-linked with *β*(1,6)-glucan, mannoproteins, other proteins, chitin, and lipids what limit the access of hydrolytic enzymes to glycosidic bonds in the chains of these macromolecules.

A significant influence of the mode of cultivation of examined yeast on the content of *β*(1,3)/(1,6)-glucan in cell wall preparations was noted. The lowest *β*-glucan concentration was determined in cell wall preparations after yeast cultivation in shake-flasks (approx. 48%). The batch fermentations allowed to obtain preparations consist with about 59.4–63.8% of discussed polysaccharide after 72 and 48 h of cultivation, correspondingly.

The applied purification procedure of cell walls contributed to the increase in the content of *β*(1,3)/(1,6)-glucan in studied preparations to 81–85%, in case of isolated from biomass propagated in flasks and biofermentor, respectively. *β*(1,3)/(1,6)-Glucan constituted to about 88.5 and 92.5% of total sugars determined in that preparations.

#### The content of alkali-insoluble and alkali-soluble polysaccharides fractions in purified *β*-glucan preparations

**Fig. 5:**
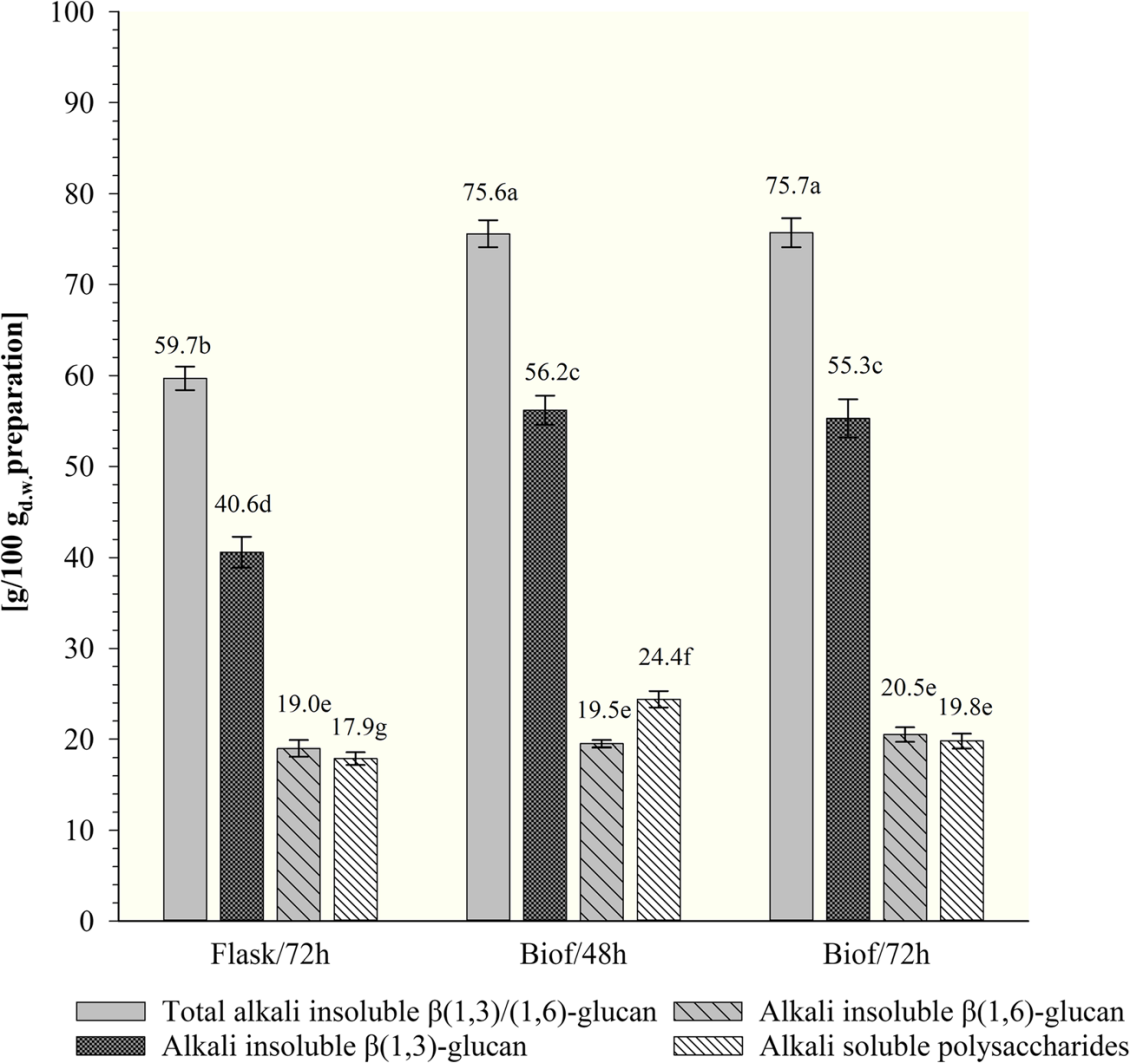
The content of alkali-insoluble and alkali-soluble polysaccharide fractions in purified β(1,3)/(1,6)-glucan preparations of *C. utilis* ATTC 9950 depending on the mode and time of cultivation in deproteinated potato juice water with 10% of glycerol (a, b, c…—mean values marked with the same letters do not differ significantly, Tukey’s test, *α* = 0.05; Flasks/72h—72 h culture in shake-flasks; Biof/48h and Biof/72h—batch fermentation cultures after 48 and 72 h, respectively). Each value represents the mean and standard deviation from three independent experiments in flask and two independent cultures in biofermentor for each of studied time of cultivation. Replicates were done on different days with different inocula stocks, using the same batch of deproteinated potato juice water as a medium

The results of purified *β*(1,3)/(1,6)-glucan characterization on the content of alkali-soluble and alkali-insoluble polysaccharides were presented on Fig. 5 and in Table 4.

**Table 4:** Percentage of *β*(1,3)- and *β*(1,6)-glucan in alkali-insoluble polysaccharides of purified *β*-glucan preparations of *C. utilis* ATCC 9950

Type of culture	*β*(1,3)-Glucan	*β*(1,6)-Glucan
	(%) of alkali-insoluble polysaccharides	
*Flask/72h	68.0^a^	31.8^b^
Biof/48h	74.3^b^	25.8^a^
Biof/72h	73.1^b^	27.1^a^

*Flasks/72h—cultivation in flask during 72 h; Biof/48h and Biof/72h—cultivations in biofermentor adequately 48 and 72 h; a, b, c…—mean values marked with the same letters do not differ significantly, Tukey’s test, *α* = 0.05. Each value represents the mean and standard deviation from three independent experiments in flask and two independent cultures in biofermentor for each of studied time of cultivation. Replicates were done on different days with different inocula stocks, using the same batch of deproteinated potato juice water as a medium

The *β*-glucan preparation isolated from the biomass propagated in shake-flasks consists with about 59.7 g of alkali-insoluble polysaccharides, while batch fermentation contributed to the increase in the content of that polysaccharide fraction to about 76 g per 100 g_d.w._ of preparation, regardless of the breeding time (Fig. 5).

In order to determine the content of particular types of *β*-glucans (*β*(1,3)- and *β*(1,6)-glucan) in the alkali-insoluble fraction, enzymatic hydrolysis was carried out using Zymolyase 20T preparation with the activity of *β*(1,3)-glucanase. The highest content of alkali-insoluble *β*(1,3)-glucan (about 55–56%) was determined in preparations isolated from yeasts grown in biofermentor (Table 4). *β*(1,3)-Glucan accounted for about 73–74% of alkali-insoluble glucans. The *β*(1,6)-glucan was present at 19.5–20.5 g/100 g_d.w._ preparation. In preparations obtained from *C. utilis* biomass propagated in flasks, the concentration of alkali-insoluble *β*(1,3)-glucan was lower (41 g/100 g_d.w._) constituting approx. 68% alkali-insoluble glucans. *β*(1,6)-Glucan accounted for 32% of alkali-insoluble fraction of discussed preparations.

## Discussion

The aim of the research was to determine the impact of mode of cultivation (shake-flask and batch fermentation) of *C. utilis* ATCC 9950 yeast in deproteinated potato juice water with the addition of glycerol (100 g/L) on the characteristics and the productivity of biomass and *β*(1,3)/(1,6)-glucan preparations. The influence of the time of yeast propagation was also under consideration in case of batch fermentation.

The process of cell walls isolation and initial purification (mechanical disintegration of cells, washing of preparations with saline solutions and with ethanol solution) contributed to the important reduction of protein content in obtained material. The purity of studied cell wall preparations was similar to commercial preparations of *β*-glucan of *Saccharomyces cerevisiae* origin described in literature (Suphantharika et al. 2003; Thammakiti et al. 2004). Purified preparations of *β*(1,3)/(1,6)-glucan were obtained by subjecting cell walls to hot water-extraction, lipid extraction with isopropyl alcohol, and enzymatic protein digestion using pronase E preparation. Obtained results considering the purity of studied *β-*glucan preparations were comparable to the ones discussed in the literature and produced by similar purification strategies (da Silva Araújo et al. 2014; Freimund et al. 2003; Liu et al. 2008; Magnani et al. 2009).

The volumetric productivity of purified *β*(1,3)(1,6)-glucan preparations and calculated output of pure *β*(1,3)(1,6)-glucan were definitely higher comparing with literature data. Pengkumsri et al. (2017) noted maximum output of *β*-glucan extracted from *S. cerevisaie* biomass on a level about 3.7 g/L. After cultivation of *Pichia pastoris* yeast on glycerol by-product fraction from biodiesel industry, the volumetric productivity of chitin-glucan complex (molar ratio 16:84) was 1.28 g/L after 45 h of fed-batch fermentation studied by Roca et al. (2012). Varelas et al. (2017) investigated the impact of glucose concentration and NaCl osmotic stress on *β*-glucan formation by wine yeast strain of *S. cerevisiae* during 192 h of fermentation. The highest *β*-glucan output (2.08 g/ L, own calculations on the basis of data presented in cited article) was noticed after 48 h fermentation in medium composed with 20% of glucose and without NaCl. The productivity of *β*-glucan reported by quoted authors was definitely lower after fermentation longer that 48 h.

The output of *β*-glucan preparations isolated from yeast depends on cell mass production as a source of that polysaccharide. The mode and time of *C. utilis* ATCC 9950 cultivation in studied medium significantly influenced cell mass production and yield. Better aeration of cultures carried in biofermentor system comparing with conditions in shake-flasks contributed to the more efficient glycerol utilization as a carbon and energy source for biosynthesis of yeast biomass components. Results presented in literature (Chiruvolu et al. 1999; Ochoa-Estopier et al. 2011; Rosma and Ooi 2006; Turcotte et al. 2010) confirm that the extend of glycerol utilization by yeast cells depends on dissolved oxygen availability in culture due to the higher degree of reduction of glycerol compared to glucose. Lee and Kim (2001) noted lower *C. utilis* biomass yield (app. 0.13 g_d.w._/g glucose equivalent) after cultivation in flasks at initial concentration of sugars amounted to 100 g/L while fed-batch fermentation with linear feeding strategy resulted in app. 0.48 g_d.w._/g glucose equivalent.

In all tested cultures, the external alkalization of medium was noted during yeast cultivation. Similar observations were reported by Neves et al. (2004) who studied glycerol transport in *S. cerevisiae* cells and explained discussed dependence by the presence of active glycerol translocation based on symport with H^+^. According to literature (Lages and Lucas 1997; Neves et al. 2004; Ochoa-Estopier et al. 2011; Rivaldi et al. 2008; van Zyl et al. 1990), active glycerol translocation is induced by yeast cultivation under high osmotic pressure, high ionic strength, or in the presence of gluconeogenic substrates (non-fermentative carbon sources, like glycerol). No significant differences in studied *C. utilis* cell mass production after 48 and 72 h of batch fermentation was noticed in spite of the availability of nitrogen and carbon sources. Chiruvolu et al. (1999) indicated that when cultivation of *Pichia pastoris* on glycerol was conducted without pH controlling, the biomass growth was limited while under constant pH conditions of 5.0 glycerol was efficiently utilized at concentrations up to 12%. On the other hand, Lages and Lucas (1997) do not confirm the significant effect of extracellular pH in the range of 3.0–7.0 on the degree of glycerol utilization by *S. cerevisiae* cells. Next studies will be directed to determine the effect of pH regulation on biomass and *β-*glucan production efficiency. Further optimization of the agitation intensity and the rate of culture aeration during batch fermentation could also improve glycerol uptake, biomass production, and *β-*glucan yield what will be under investigation. Gancedo et al. (1968) underline that especially in *C. utilis* aeration is a requirement for growth on glycerol while the growth on glucose is possible under nearly anaerobic conditions.

Klein et al. (2017) and Turcotte et al. (2010) described that when glycerol was used as a carbon source for *S. cerevisiae* cultivation, the expressions of genes encoding proteins related to mitochondrial function an energy metabolism were highly up-regulated but also enzymes responsible for gluconeogenesis and carbohydrate storage. The products of glycerol oxidation could enter into gluconeogenesis pathway in cytosol (Klein et al. 2017; Rivaldi et al. 2008; Turcotte et al. 2010). In the tested culture medium based on DPJW with the addition of 10% glycerol, the yeast cells were subjected to osmo-stress. Glycerol is a compound that increases the osmotic pressure more intensively than glucose as solute. According to Ochoa-Estopier et al. (2011) in media with an addition of 20 to 150 g/L of glycerol, the osmotic pressure rises from 0.65 to 1.00 Osm/kg. Under elevated extracellular osmotic pressure, the high osmolarity glycerol (HOG) pathway increases the de novo glycerol synthesis and/or its uptake from environment, and limits its lost from the cells (Duškova et al. 2015). Uncontrolled intracellular glycerol accumulation could result in disturbing normal osmotic balance and substrate accelerated cell death that is why glycerol catabolism is needed (Klein et al. 2017; Tao et al. 1999). Beese et al. (2009) found that the increased turgor pressure provokes the cells of *S. cerevisiae* yeast to fortify cell wall by polysaccharides synthesis what made the structure more resistant to zymolyase activity. Other authors (Borovicova et al. 2016; Dalonso et al. 2015; García et al. 2009; Gow et al. 2017; Kopecká et al. 1991; Varelas et al. 2017; Xu et al. 2016) also indicate yeast cell wall remodeling strategy and *β*-glucan synthesis as adaptation mechanisms for development under increased osmotic pressure. Babazadeh et al. (2017) studied at the first time the response to hyperosmotic stress in respiring *S. cerevisiae* cells cultivated in medium with non-fermentable carbon source (ethanol). They observed significant enrichment for up-regulated genes encoding glucan metabolic processes, among other genes. Osmotic stress inducts signaling pathways of mitogen-activated kinases like MAP Hog1 and Slt2 kinases in yeast cells (Bermejo et al. 2008; Duškova et al. 2015). The Stl2 kinase pathway is responsible for cytoskeleton strengthening of yeast cells activating regulatory proteins enzymes responsible for *β*-glucan and chitin synthesis (Hohmann 2002). The *FKS2* gene encoding *β*(1,3)-glucan synthase complex is transcribed in response to yeast growth in media with the addition of carbon sources other than glucose, as well as in response to stress factors affecting the yeast cell wall (Smits et al. 2001). Overexpression of *FKS2* genes contributes to the increase in *β*-glucan content in yeast cell walls (Smits et al. 2001; Xu et al. 2016).

**Fig. 6:**
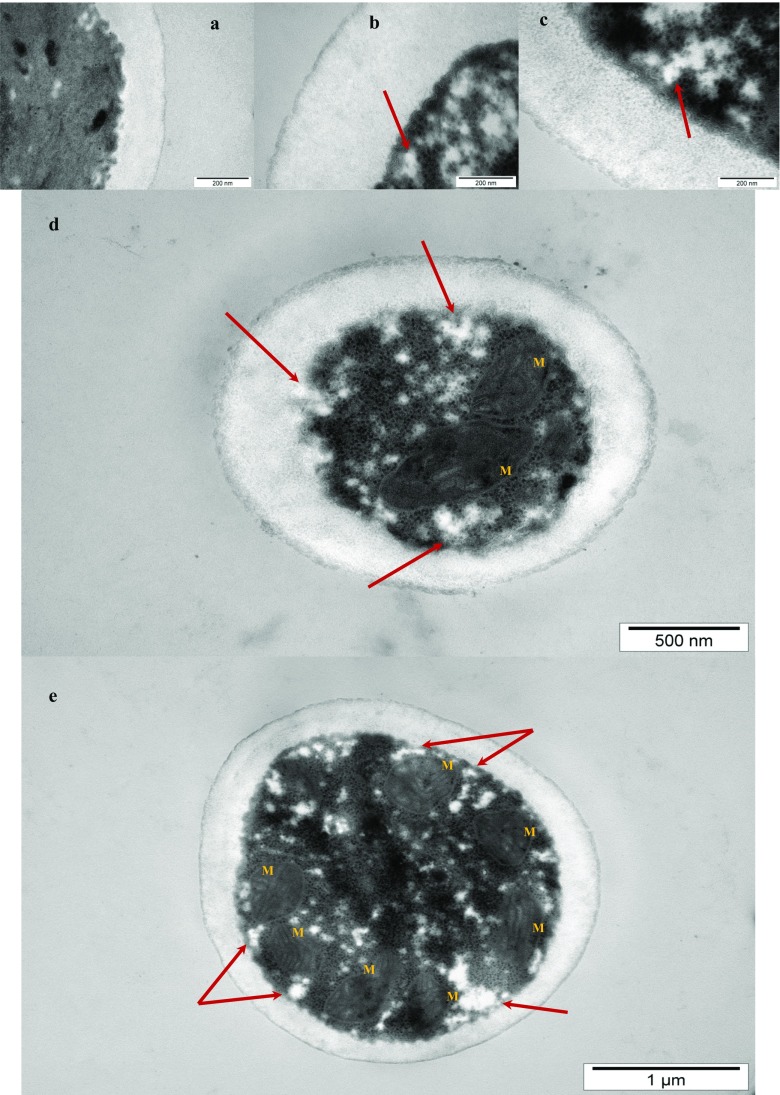
Exemplary microscopic (TEM) photographs of the yeast *C. utilis* ATCC 9950 strain cell walls on YPD medium (**a**) and DPJW with 10% of glycerol (**b–e**); *arrows* indication of possible *β-*glucan deposits; *M* mitochondria

On Fig. 6a–e, there are presented exemplary microscopic (TEM) photographs of cell walls and cells of studied *C. utilis* ATCC 9950 strain on YPD medium (Fig. 6a) and DPJW with 10% of glycerol (Fig. 6b–e). Definitely thicker cell walls were visible after yeast cultivation on DPJW with 10% glycerol. Interestingly, in cytoplasm of tested yeast from the discussed cultivation medium, irregular, electron-lucent material was visible (Fig. 6b–e, indicated by arrows). This material was located near to the cell wall and mitochondria and looks like being introduced into the structure of discussed organellum. Tkacz (1992) indicated that *β*-glucan appeared as a disorganized microfibryllar mass by electron microscopy when the biosynthesis of that polysaccharide was studied using separated glucan synthase complex. According to literature (Schomburg and Dörte 1996), glycerol may activate *β*(1,3)-glucan synthase. It prompt us to put the hypothesis that discussed material would be depots of insoluble *β-*glucan (Fig. 6b–e, indicated by arrows) which were formed from glucose occurred in gluconeogenesis. Considering the location of the mitochondria close to the cellular membrane (Fig. 6d, e), the glucan synthesis from products of glycerol oxidation was possible and energy-explanatory. Up-regulation of this process could result from osmo-stress conditions and glucose depletion (Beese et al. 2009; Babazadeh et al. 2017; Levin 2011; Turcotte et al. 2010). This could explain high sugars and *β*(1,3)/(1,6)-glucan content in the biomass and cell walls of studied yeast, indicating glycerol utilization in *β*-glucan synthesis. Proposed claim is obviously discursive and needs for further research to confirm the assumption. Immunocytology would be needed as an example. According to available literature data, *β*-glucan synthase is placed in the cell membrane, however towards the cytoplasm (Firon et al. 2004; Levin 2011; Lipke and Ovalle 1998; Papaspyridi et al. 2018). The *β*-glucan chains (up to 1500 glucose monomers) are synthesized in the cytosol and then are transferred to the periplasmic space by a transmembrane enzyme. Further modifications of the polimer structure, like side branching addition, take place in the periplasmic space (Papaspyridi et al. 2018). Nevertheless, discussed enzymatic complex is still not fully characterized. Recently, Papaspyridi et al. (2018) summarized data published previously on glucan synthase biology. Quoted authors underlined that the key enzyme in *β*(1,3)-glucan synthesis is regarded as a largely unexplored biotechnological tool and the mechanism of *β-*glucan synthesis is a topic of ongoing investigations. At the same time there is no data considering *β*(1,3)-glucan synthesis under conditions similar to described in current work.

In yeast cell wall, there are identified alkali-insoluble and alkali-soluble *β*-glucan fractions. The insolubility of yeast *β-*glucan in bases is explained by the connection of that polysaccharides with chitin (Klis et al. 2002) or results from the degree of polymerization and branching or glycoside bonds location in the polysaccharide (Ha et al. 2002; Huang and Li 2012; Kath and Kulicke 1999; Mantovani et al. 2008; Šandula et al. 1999). For the construction of a resistant cell wall, *β*(1,6)-glycosidic side branches are added, connecting several *β*1,3)-glucan chains together (Papaspyridi et al. 2018).

Alkali-insoluble *β*-glucan predominates in cell wall of *S. cerevisiae* yeast (Kath and Kulicke 1999). It was also the main polysaccharide fraction stated in studied *β*-glucan preparations of *C. utilis* origin. In turn, Nguyen et al. (1998) indicated that it is a strain-dependent factor. Stimulation of alkali-insoluble *β*(1,3)-glucan synthesis in batch fermentation, regardless of the breeding time, could be a consequence of more efficient gluconeogenesis discussed above. It could be also associated with the stress caused by shearing forces during intensive mixing of the *C. utilis* culture in the biofermentor. The *β*-glucan polymer determines the mechanical strength of yeast cell walls (Klis et al. 2002). Hartland et al. (1994) described the alkali-soluble *β*-glucan as a precursor of *β*-glucan insoluble in bases. This may explain the higher content of alkali-soluble polysaccharides stated in tested *β*-glucan preparations after 48 h of batch fermentation comparing with obtained from *C. utilis* biomass cultivated during 72 h.

Investigated preparations differed in the content of alkali-insoluble *β*(1,6)-glucan also. The polymer of *β*(1,6)-glucan mediates cross-links between mannoproteins and *β*(1,3)-glucan in yeast cell walls (Smits et al. 2001). In *β*-glucan preparations produced from biomass propagated in flasks, a higher content of proteins was determined, presumably constituting a group of proteins integrally incorporated into the cell wall structure, and therefore more difficult to remove during purification process. The level of culture oxidation influences on protein composition of yeast cell walls (Klis et al. 2006). This would explain the higher content of *β*(1,6)-glucan in preparations isolated from biomass cultivated in shake-flask comparing with preparations obtained after batch fermentation. However, Naruemon et al. (2013) observed mannoproteins decrease in yeast cell walls with simultaneous *β*(1,6)-glucan increase, implying more branching *β*-glucan structure probably important to increase the integrity of glucan matrix and cell wall strength under growth conditions studied by quoted authors.

Summarizing, it was the first try to scale up the cultivation of *C. utilis* ATCC 9950 in waste deproteinated potato juice water with the addition of glycerol directed to *β*(1,3)/(1,6)-glucan production. The mode of yeast cultivation influenced the chemical characteristic of biomass and obtained *β*-glucan preparations. The biomass consists with lower protein content and higher sugars concentration after cultivation in biofermentor system which is advantageous in obtaining purified *β-*glucan preparations. The highest cell mass production parameters and *β*-glucan preparations of studied *C. utilis* strain were achieved after 48 h incubation in batch fermentation. Produced *β*-glucan preparations were characterized by 81–85 purity after shake-flasks and biofermentor cultivation, respectively. Batch fermentation promoted biosynthesis of alkali-insoluble *β*(1,3)-glucan fraction, decreasing the content of *β*(1,6)-glucan. Further optimization of culture conditions is needed, including agitation intensity and the rate of culture aeration during batch fermentation to improve glycerol uptake by the yeast under investigation. Next studies will be also directed to determine the effect of pH stabilization on the rate of glycerol utilization and biomass production efficiency as well as *β*-glucan output.

## References

[CR1] Aguilar-Uscanga B, François JM (2003). A study of the yeast cell wall composition and structure in response to growth conditions and mode of cultivation. Lett Appl Microbiol.

[CR2] Auinger A, Riede L, Bothe G, Busch R, Gruenwald J (2013). Yeast (1,3)-(1,6)-beta-glucan helps to maintain the body’s defence against pathogens: a double-blind, randomized, placebo-controlled, multicentric study in healthy subjects. Eur J Nutr.

[CR3] Babazadeh R, Lahtvee P-J, Adiels CB, Goksör M, Nielsen JB, Hohmann S (2017). The yeast osmostress response is carbon source dependent. Sci Rep.

[CR4] Bacha U, Nasir M, Iqbal S, Anjum AA (2017). Nutraceutical, anti-inflammatory, and immune modulatory effects of *β*-glucan isolated from yeast. Biomed Res Int.

[CR5] Baert K, De Geest BG, De Greve H, Cox E, Devriendt B (2016). Duality of β-glucan microparticles: antigen carrier and immunostimulants. Int J Nanomedicine.

[CR6] Batbayar S, Lee DH, Kim HW (2012). Immunomodulation of fungal *β*-glucan in host defense signaling by dectin-1. Biomol Ther.

[CR7] Beese SE, Negishi T, Levin DE (2009). Identification of positive regulators of the yeast Fps1 glycerol channel. PLoS Genet.

[CR8] Bekatorou A, Psarianos C, Koutinas AA (2006). Production of food grade yeasts. Food Technol Biotechnol.

[CR9] Bermejo C, Rodríguez E, García R, Rodríguez-Peña JM, Rodríguez de la Concepción ML, Rivas C, Arias P, Nombela C, Posas F, Arroyo J (2008). The sequential activation of the yeast HOG and SLT2 pathways is required for cell survival to cell wall stress. Mol Biol Cell.

[CR10] Borchani C, Fonteyn F, Jamin G, Destain J, Willems L, Paquot M, Blecker C, Thonart P (2016). Structural characterization, technological functionality, and physiological aspects of fungal β-D-glucans: a review. Crit Rev Food Sci Nutr.

[CR11] Borovikova Diana, Teparić Renata, Mrša Vladimir, Rapoport Alexander (2016). Anhydrobiosis in yeast: cell wall mannoproteins are important for yeastSaccharomyces cerevisiaeresistance to dehydration. Yeast.

[CR12] Bzducha-Wróbel A, Kieliszek M, Błażejak S (2013). Chemical composition of the cell wall of probiotic and brewer’s yeast in response to cultivation medium with glycerol as a carbon source. Eur Food Res Technol.

[CR13] Bzducha-Wróbel A, Błażejak S, Molenda M, Reczek L (2015). Biosynthesis of *β*(1,3)/(1,6)-glucans of cell wall of the yeast *Candida utilis* ATCC 9950 strains in the culture media supplemented with deproteinated potato juice water and glycerol. Eur Food Res Technol.

[CR14] Chiruvolu V, Eskridge K, Cregg J, Meagher M (1999). Effect of glycerol concentration and pH on growth of recombinant *Pichia pastoris* yeast. Appl Biochem Biotechnol.

[CR15] Da Silva Araújo VB, DE Melo ANF, Costa AG, Castro-Gomez RH, Madruga MS, de Souza EL, Magnani M (2014). Followed extraction of β-glucan and mannoproteiny from spent brewer’s yeast (*Saccharomyces uvarum*) and application of the obtained mannoproteiny as a stabilizer in mayonnaise. Innovative Food Sci Emerg Technol.

[CR16] Dalonso N, Goldman GH, Gern RM (2015). β-(1→3),(1→6)-Glucans: medicinal activities, characterisation, biosynthesis and new horizons. Appl Microbiol Biotechnol.

[CR17] Duškova M, Borovikova D, Herynkova P, Rapoport A, Sychrova H (2015). The role of glicerol transporters in yeast cells in various physiological and stress conditions. FEMS Microbiol Lett.

[CR18] Dzwonkowski W (2012). Perspektywy rynku skrobi i produkcji ziemniaków skrobiowych w kontekście zmian wspólnej polityki rolnej. Biul IHAR.

[CR19] Ferrari MD, Bianco R, Froche C, Loperena ML (2001). Baker’s yeast production from molasses/cheese whey mixtures. Biotechnol Lett.

[CR20] Firon A, Lesage G, Bussey H (2004). Integrative studies put cell wall synthesis on the yeast functional map. Curr Opin Microbiol.

[CR21] Freimund S, Sauter M, Käppeli O, Dutler H (2003). A new non-degrading isolation process for 1,3-β-D-glucan of high purity from baker’s yeast *Saccharomyces cerevisiae*. Carbohydr Polym.

[CR22] Gancedo C, Gancedo JM, Sols A (1968). Glycerol metabolism in yeasts. Pathways of utilization and production. Eur J Biochem.

[CR23] García R, Rodríguez-Peña JM, Bermejo C, Nombela C, Arroyo J (2009). The high osmotic response and cell wall integrity pathways cooperate to regulate transcriptional responses to zymolyase-induced cell wall stress in *Saccharomyces cerevisiae*. J Biol Chem.

[CR24] Gow NAR, Latge JP, Munro C (2017) The fungal cell wall: structure, biosynthesis, and function. Microbiol Spectr. 10.1128/microbiolspec.FUNK-0035-2016 10.1128/microbiolspec.funk-0035-2016 PMC11687499 28513415

[CR25] Ha CH, Lim KH, Kim YT, Lim ST, Kim CW, Chang HI (2002). Analysis of alkali-soluble glucan produced by *Saccharomyces cerevisiae* wild-type and mutants. Appl Microbiol Biotechnol.

[CR26] Hartland RP, Vermeulen CA, Klis FM, Sietsma JH, Wessels JG (1994). The linkage of (1-3)-beta-glucan to chitin during cell wall assembly in *Saccharomyces cerevisiae*. Yeast.

[CR27] Hohmann S (2002). Osmotic adaptation in yeast-control of the yeast osmolyte system. Int Rev Cytol.

[CR28] Huang G, Li J (2012). Efficient preparation of alkali-insoluble (1→3)-*β*-D-glucan. Int J Food Sci Nutr.

[CR29] Jaehrig SC, Rohn S, Kroh LW, Wildenauer FX, Lisdat F, Fleischer LG, Kurz T (2008). Antioxidative activity of (1→3),(1→6)-*β*-D-glucan from *Saccharomyces cerevisiae* grown on different media. LWT.

[CR30] Kath F, Kulicke WM (1999). Mild enzymatic isolation of mannan and glucan from yeast *Saccharomyces cerevisiae*. Angew Makromolek Chem.

[CR31] Klein M, Swinnen S, Thevelein JM, Nevoigt E (2017). Glycerol metabolism and transport in yeast and fungi: established knowledge and ambiguities. Environ Microbiol.

[CR32] Klis FM, Mol P, Hellingwerf K, Brul S (2002). Dynamics of cell wall structure in *Saccharomyces cerevisiae*. FEMS Microbiol Rev.

[CR33] Klis Frans M., Boorsma Andre, De Groot Piet W. J. (2006). Cell wall construction inSaccharomyces cerevisiae. Yeast.

[CR34] Kopecká M, Gabriel M, Ol N, Svoboda A, Venkov PV (1991). Cell surface structures in osmotically fragile mutants of *Saccharomyces cerevisiae*. J Gen Microbiol.

[CR35] Kowalczewski P, Celka K, Białas W, Lewandowicz G (2012). Antioxidant activity of potato juice. Acta Sci Pol Technol Aliment.

[CR36] Kurcz A, Błażejak S, Kot AM, Bzducha-Wróbel A, Kieliszek M (2018). Application of industrial wastes for the production of microbial single-cell protein by fodder yeast *Candida utilis*. Waste Biomass Valor.

[CR37] Lages F, Lucas C (1997). Contribution to the physiological characterization of glycerol active uptake in *Saccharomyces cerevisiae*. Biochim Biophys Acta.

[CR38] Lee BK, Kim JK (2001). Production of *Candida utilis* biomass on molasses in different culture types. Aquac Eng.

[CR39] Levin DE (2011). Regulation of cell wall biogenesis in *Saccharomyces cerevisiae*: the cell wall integrity signaling pathway. Genetics.

[CR40] Lipke PN, Ovalle R (1998). Cell wall architecture in yeast: new structure and new challenges. J Bacteriol.

[CR41] Liu XY, Wang Q, Cui SW, Liu HZ (2008). A new isolation method of β-D-glucans from spent yeast *Saccharomyces cerevisiae*. Food Hydrocoll.

[CR42] Magnani M, Calliari CM, de Macedo JFC, Mori MP, de Syllos Cólus IM, Casto-Gomez RJH (2009). Optimized methodology for extraction of (1→3)(1→6)-β-D-glucan from *Saccharomyces cerevisiae* and in vitro evaluation of the cytotoxicity and genotoxicity of the corresponding carboxymethyl derivative. Carbohydr Polym.

[CR43] Mantovani MS, Bellini MF, Angeli JP, Oliveira RJ, Silva AF, Ribeiro LR (2008). Beta-glucans in promoting health: prevention against mutation and cancer. Mutat Res.

[CR44] Milchert E, Goc W, Lewandowski G, Myszkowski J (1995). Dehydrochlorination of glycerol dichlorohydrin to epichlorohydrin. Chem Pap.

[CR45] Naruemon M, Romanes S, Cheunjit P, Xiao H, McLandsborough LA, Pawadee M (2013). Influence of additives on *Saccharomyces cerevisiae β*-glucan production. Int Food Res J.

[CR46] Neves L, Oliveira R, Lucas C (2004). Yeast orthologues associated with glycerol transport and metabolizm. FEMS Yeast Res.

[CR47] Nguyen TH, Fleet GH, Rogers PL (1998). Composition of the cell walls of several yeast species. Appl Microbiol Biotechnol.

[CR48] Ochoa-Estopier A, Lesage J, Gorret N, Guillouet SE (2011). Kinetic analysis of a *Saccharomyces cerevisiae* strain adapted for improved growth on glycerol: implications for the development of yeast bioprocesses on glycerol. Bioresour Technol.

[CR49] Papaspyridi L-M, Zerva A, Topakas E (2018). Biocatalytic synthesis of fungal *β*-glucans. Catalysts.

[CR50] Pengkumsri N, Sivamaruthi BS, Sirilun S, Peerajan S, Kesika P, Chaiyasut K, Chaiyasut C (2017). Extraction of *β*-glucan from *Saccharomyces cerevisiae*: comparison of different extraction methods and in vivo assessment of immunomodulatory effect in mice. Food Sci Technol.

[CR51] Richter J, Svozil V, Král V, Dobiášová LR, Vetlicka V (2015). β-Glucan affects mucosal immunity in children with chronic respiratory problems under physical stress: clinical trials. Ann Transl Med.

[CR52] Rivaldi JD, Sarrouh BF, da Silva SS (2008). Development of biotechnological processes using glycerol from biodiesel production. Current research topics in applied microbiology and microbial biotechnology.

[CR53] Roca C, Chagas B, Farinha I, Freitas F, Mafra L, Aguiar F, Oliveira R, Reis MAM (2012). Production of yeast chitin-glucan complex from biodiesel industry byproduct. Process Biochem.

[CR54] Rosma A, Ooi KI (2006). Production of *Candida utilis* biomass and intracellular protein content; effect of agitation speed and aeration rate. Malays J Microbiol.

[CR55] Samuelsen AB, Schrezenmeir J, Knutsen SH (2014). Effects of orally administered yeast-derived beta-glucans: a review. Mol Nutr Food Res.

[CR56] Šandula J, Kogan G, Kačuráková M, Machová E (1999). Microbial (1→3)-*β*-D-glucans, their preparation, physico-chemical characterization and immunomodulatory activity. Carbohydr Polym.

[CR57] Schomburg D, Dörte S (1996). 1,3-beta-Glucan synthase. Enzyme handbook 12.

[CR58] Shao Y, Wang Z, Tian X, Guo Y, Zhang H (2016). Yeast *β*-d-glucans induced antimicrobial peptide expressions against *Salmonella* infection in broiler chickens. Int J Biol Macromol.

[CR59] Sitepu IR, Garay LA, Sestric R, Levin D, Block DE, German JB, Boundy-Mills KL (2014). Oleaginous yeasts for biodiesel: current and future trends in biology and production. Biotechnol Adv.

[CR60] Smits GJ, van den Ende H, Klis FM (2001). Differential regulation of cell wall biogenesis during growth and development in yeast. Microbiology.

[CR61] Stier H, Ebbeskotte V, Gruenwald J (2014). Immune-modulatory effect of dietary yeast beta-1,3/1,6-D-glucan. Nutr J.

[CR62] Suphantharika M, Khunrae P, Thanardkit P, Verduyn C (2003). Preparation of spent brewer’s yeast β-glucans with a potential application as an immunostimulant for black tiger shrimp *Penaeusmonodon*. Bioresour Technol.

[CR63] Tao W, Deschenes RJ, Fassler JS (1999). Intracellular glycerol levels modulate the activity of Sln1p, a *Saccharomyces cerevisiae* two-component regulator. J Biol Chem.

[CR64] Thammakiti S, Suphantharika M, Phaesuwan T, Verduyn C (2004). Preparation of spent brewer’s yeast β-glucans for potential application in the food industry. Int J Food Sci Technol.

[CR65] Tkacz JS, Sutcliffe JA, Georgopapadakou NH (1992). Glucan biosynthesis in fungi and its inhibition. Emerging targets in antibacterial and antifungal chemotheraphy.

[CR66] Turcotte B, Liang XB, Robert F, Soontorngun N (2010). Transcriptional regulation of nonfermentable carbon utilization in budding yeast. FEMS Yeast Res.

[CR67] van Zyl PJ, Kilian SG, Prior BA (1990). The role of an active transport mechanism in glycerol accumulation during osmoregulation by *Zygosaccharomyces rouxii*. Appl Microbiol Biotechnol.

[CR68] Varelas V, Sotiropoulou E, Karambini X, Liouni M, Nerantzis ET (2017). Impact of gluycose concentration and NaCl osmotic stress on yeast cell wall *β*-D-glucan formation during anaerobic fermentation process. Fermentation.

[CR69] Wang Q, Sheng X, Shi A, Hu H, Yang Y, Liu L, Fei L, Liu H (2017). β-Glucans: relationships between modification, conformation and functional activities. Molecules.

[CR70] Xu S, Zhang GY, Zhang H, Kitajima T, Nakanishi H, Gao XD (2016). Effects of Rho1, a small GTPase on the production of recombinant glycoproteins in *Saccharomyces cerevisiae*. Microb Cell Factories.

[CR71] Zhu F, Du B, Xu B (2016). A critical review on production and industrial applications of beta-glucans. Food Hydrocoll.

